# Development of a high efficiency integration system and promoter library for rapid modification of *Pseudomonas putida* KT2440^☆^

**DOI:** 10.1016/j.meteno.2017.04.001

**Published:** 2017-04-15

**Authors:** Joshua R. Elmore, Anna Furches, Gara N. Wolff, Kent Gorday, Adam M. Guss

**Affiliations:** Biosciences Division, Oak Ridge National Laboratory, One Bethel Valley Road, Oak Ridge, TN 37831, USA

**Keywords:** Pseudomonas putida, Genetic engineering, Promoter library, Site-specific recombinase, Gene expression

## Abstract

*Pseudomonas putida* strains are highly robust bacteria known for their ability to efficiently utilize a variety of carbon sources, including aliphatic and aromatic hydrocarbons. Recently, *P. putida* has been engineered to valorize the lignin stream of a lignocellulosic biomass pretreatment process. Nonetheless, when compared to platform organisms such as *Escherichia coli*, the toolkit for engineering *P. putida* is underdeveloped. Heterologous gene expression in particular is problematic. Plasmid instability and copy number variance provide challenges for replicative plasmids, while use of homologous recombination for insertion of DNA into the chromosome is slow and laborious. Further, most heterologous expression efforts to date typically rely on overexpression of exogenous pathways using a handful of poorly characterized promoters. To improve the *P. putida* toolkit, we developed a rapid genome integration system using the site-specific recombinase from bacteriophage Bxb1 to enable rapid, high efficiency integration of DNA into the *P. putida* chromosome. We also developed a library of synthetic promoters with various UP elements, −35 sequences, and −10 sequences, as well as different ribosomal binding sites. We tested these promoters using a fluorescent reporter gene, mNeonGreen, to characterize the strength of each promoter, and identified UP-element-promoter-ribosomal binding sites combinations capable of driving a ~150-fold range of protein expression levels. An additional integrating vector was developed that confers more robust kanamycin resistance when integrated at single copy into the chromosome. This genome integration and reporter systems are extensible for testing other genetic parts, such as examining terminator strength, and will allow rapid integration of heterologous pathways for metabolic engineering.

## Highlights

• BxB1 integrase catalyzes site-specific DNA integration into *P. putida* chromosome.
• Promoter library (−35/−10 variants) covers a 72-fold range of protein expression.
• Expression can be further tuned by 2-fold in *P. putida* with RBS and UP-elements.

## 1 Introduction

Given the world's finite amount of fossil fuels, the need for renewable sources of fuels and commodity chemicals is becoming increasingly important. To address this issue, much work has gone into the production of these chemicals from biological sources. The majority of research to date has focused on the conversion of cellulosic biomass from plants into value added chemicals. However, recent reports have shown that biorefineries will need to valorize lignin streams to be economically viable (Davis et al., 2013). Lignin comprises 15–40% of terrestrial plant biomass (Ragauskas et al., 2014). Unlike cellulosic biomass, which is largely comprised of homogenous mixture of polysaccharides, lignin is a heterogeneous and interlinked polymer comprised of aromatic compounds which has limited its use in biorefineries. Currently, lignin streams in most biorefineries are slated for combustion for the generation of process heat and electricity. Several groups have used a combination of chemical and/or biological means to depolymerize lignin, and some have taken advantage of aromatic-consuming bacteria, such as *Pseudomonas putida* species, to transform lignin streams into value added chemicals (Linger et al., 2014, Rahimi et al., 2014, Vardon et al., 2015, Zakzeski et al., 2012).

A major limiting factor for rapid metabolic engineering of *Pseudomonas putida* is the availability of tools for sophisticated genetic engineering. The primary tools currently used for directed genetic engineering in *P. putida* KT2440, replicating plasmids such as derivatives of the pBBR family of plasmids (Kovach et al., 1995) and homologous recombination-based gene replacement technologies (Johnson and Beckham, 2015, Schafer et al., 1994), have many shortcomings. Replicating plasmids typically require constant selection for maintenance, have highly variable copy number (Lin-Chao and Bremer, 1986, Paulsson and Ehrenberg, 2001), and often come with a fitness cost (Mi et al., 2016). Current homologous recombination-based technologies rely on inefficient plasmid integration by host DNA repair enzymes, leading to low transformation rates. Recombination of these plasmids by single cross-over events leads to genome structures that are inherently unstable, allowing deletion or integration of DNA sequences, but simultaneously makes them unsuitable for rapid prototyping. In an attempt to address some of these shortcomings, *P. putida* genetic engineering tools have been expanded to include oligonucleotide (Aparicio et al., 2016), λ Red/Cre (Luo et al., 2016), and meganuclease (Martinez-Garcia and de Lorenzo, 2011) recombineering technologies.

Site-specific recombinases are enzymes that catalyze recombination between two specific sequences of DNA (for a review see (Brown et al., 2011)). Recombinases Flp and Cre are commonly used in molecular genetics, but these enzymes recognize identical sites and perform reversible recombination between the sites. Serine recombinases such as ΦC31 integrase, on the other hand, natively catalyze unidirectional phage genome integration through the recombination between *attP* (phage genome) and *attB* (bacterial genome) sequences, generating distinct *attL* and *attR* sequences in the process. Unlike the unidirectional λ phage integrase from *E. coli* that requires the *E. coli* protein IHF for recombination, these serine recombinases do not require any host factors and have been used for chromosomal insertion of heterologous DNA in organisms across the tree of life (Brown et al., 2011, Guss et al., 2008, Keravala et al., 2006, Siuti et al., 2014, Thomson et al., 2012, Xu and Brown, 2016). In addition to ΦC31 integrase, other serine recombinases have been identified and characterized, including the determination of the corresponding *attB* and *attP* sites enabling these systems to also be developed as genetic tools (Brown et al., 2011).

In other organisms, tuning protein expression can be very important for achieving increased yields from engineered pathways (Alper et al., 2005, Jones et al., 2015, Xu et al., 2013), but there are no well-characterized promoter or RBS libraries in *P. putida* to enable rational tuning of protein expression. Previous engineering efforts in *P. putida* have primarily relied on the lac family promoters (lac, lacUV5, tac, trc) from *E.coli* (Borrero-de Acuna et al., 2014, Johnson and Beckham, 2015, Meijnen et al., 2008, Meijnen et al., 2009, Nielsen et al., 2009, Vardon et al., 2015), with a few using native promoters such as Pm (Gemperlein et al., 2016), rrn (Wang et al., 2010), or PP_1099 promoter (Lin et al., 2016). The relative expression of the lac-family promoters in *P. putida* remains unknown, and the use of native promoters increases the likelihood of cryptic promoter regulation. To address these gaps in genetic tools, we tested serine recombinases to develop a high efficiency integration system to characterize a library of genetic elements to enable rational tuning of protein expression in *P. putida*.

## 2 Materials and methods

### 2.1 Plasmid construction

Phusion® High Fidelity Polymerase (Thermo Scientific) and primers synthesized by Integrated DNA Technologies (IDT) or Eurofins Genomics were used in all PCR amplifications for plasmid construction. Plasmids were constructed using NEBuilder® HiFi DNA Assembly Master Mix (New England Biolabs – NEB) or T4 DNA ligase (NEB) according to manufacturer's instructions. Plasmids were transformed into either competent Top10 (Life Technologies), NEB 5-alpha F’I^q^ (NEB), or Epi400 (Epicentre Biotechnologies) *Escherichia coli* according to manufacturer's instructions. Transformants were selected on LB (Miller) agar plates containing 50 mg/L kanamycin sulfate for selection and incubated at 37 °C. Plasmids were constructed using a combination of ligation of phosphorylated oligonucleotides, DNA synthesis by GenScript and IDT and Gibson Assembly. Sequences of all plasmids were confirmed using Sanger sequencing performed by GenScript or Eurofins Genomics. Annotated plasmid sequences are provided in Supplemental File 1.

### 2.2 Pseudomonas putida transformation and strain construction

#### 2.2.1 Construction of hsdR deletion and integrase replacement strains by homologous recombination

*P. putida* KT2440 was used as a wild-type parent strain for all strains. *hsdR* deletion and integrase replacement strains were constructed using the pK18mob-sacB kanamycin resistance/sucrose resistance selection/counter-selection marker system (Marx, 2008) as described in detail previously (Johnson and Beckham, 2015). Briefly, transformations were carried out by electroporation of ~500–1000 ng of plasmid DNA into KT2440 cells. Transformed colonies were selected by growth overnight at 30 °C on LB agar medium with 50 µg/mL kanamycin sulfate. Resulting transformed colonies were single colony purified and incubated overnight at 30 °C on LB agar medium containing 50 µg/mL kanamycin sulfate to eliminate residual untransformed cells from being transferred. For counter-selection, colonies were streaked onto YT+25% sucrose plates (10 g/L yeast extract, 20 g/L tryptone, 250 g/L sucrose and 18 g/L agar) and incubated overnight at 30 °C. Resulting colonies typically have recombined to remove *sacB* from the genome, as its expression causes a significant growth defect in the presence of sucrose. However, cells containing *sacB* can grow very slowly in the presence of sucrose, so colonies were streaked for isolation again on YT+25% sucrose plates and incubated at 30 °C overnight to reduce the possibility of transferring cells in which *sacB* remains in the genome. The resulting colonies (typically 20) were cultured in LB broth overnight at 30 °C and screened for either the deletion of *hsdR* or replacement of *hsdR* with a P_tac-_integrase/*attB* cassette by colony PCR and kanamycin sensitivity. *P. putida* integrase strains are listed in Table 1.

**Table 1:** *P. putida* strains constructed for bacteriophage serine integrase testing.

**Strain**	**Integrase/attB**	**Genotype**
JE1646	None	∆*hdsR*
JE1644	FBT1	∆*hdsR*::Ptac:*ΦBT1int-attB*
JE1645	FC1	∆*hdsR*::Ptac:*ΦC1int-attB*
JE1643	RV	∆*hdsR*::Ptac:*RVint-attB*
JE90	BxB1	∆*hdsR*::Ptac:*BxB1int-attB*

#### 2.2.2 Plasmid transformation assays

**Fig. 1:**
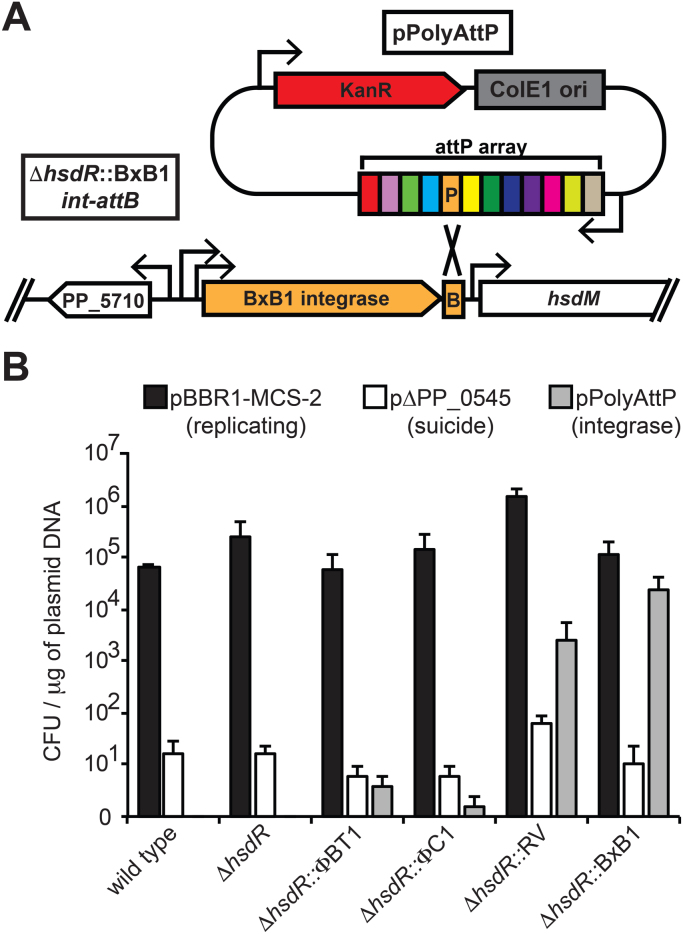
Serine integrase activity in *Pseudomonas putida* KT2440. **(A)** Graphic representation of integrase-catalyzed between the BxB1-attP (P) of pPolyAttP and the BxB1-attB (B) site in the genome of Δ*hsdR*::*BxB1int-attB*, with arrows representing putative promoter sequences. **(B)** Chart of plasmid transformation efficiency in 6 strains with a replicating (black), suicide (white), or integrase target plasmid (gray), with error bars representing the standard deviation in 3 replicate assays.

Plasmid transformation assays were performed using a variation of the transformation procedure described in Section 2.2.1. 600 ng of each plasmid was transformed into either wild-type, JE90, JE1643-1646 (Table 1) electrocompetent cells. Following electroporation, cells were resuspended in 950  μl SOC, transferred to a microfuge tube, and incubated at 30 C to allow recovery. Due to the low efficiency of homologous recombination, all of the recovery volume was plated for pΔPP_0545. For the remaining plasmids, several fractions of the recovery cultures were plated and enumerated. For Fig. 1 assays, all samples were plated on LB agar supplemented with 50 μg/mL kanamycin sulfate, incubated for 24 h at 30 °C, then enumerated. For Fig. 3 assays, samples were plated on LB agar media supplemented with 15 or 50 μg/mL kanamycin sulfate, incubated for 16 h at 30 °C, enumerated, incubated for an additional 24 h (total 40 h) again at 30 °C, and enumerated a second time. Correct insertion of DNA into the *attB* site was confirmed using multiplex PCR as described in Supplemental Figs. 2 and 3. Analogous to the *E. coli* CRIM system (Haldimann and Wanner, 2001b), multiplex PCR using a 4 primer set (primers 1–4 Supplemental Figs. 2 and 3) were used to verify plasmid integration. Primers 1 and 2 flank the *attB* and primers 3 and 4 flank the *attP*. After site-specific integration, primers 1 and 4 flank the resulting *attL* and primers 2 and 3 flank the resulting *attR*. The sizes of the *attB*/*attP*/*attL*/*attR* PCR products are all different and can be resolved on an agarose gel. If three bands representing *attL*, *attR*, and *attP* are present, it indicates that in addition to integration via the phage integrase, there is an additional copy of the plasmid present. This second copy likely inserted via homologous recombination into the first insertion or integration of a concatamer from *E. coli*, preserving an *attP* site.

#### 2.2.3 Construction fluorescent reporter strains

Fluorescent reporter strains for promoter and ribosomal binding site characterization were constructed by a variant of the transformation and strain construction procedure described previously (Johnson and Beckham, 2015). Electrocompetent JE90 cells were transformed with 200–400 ng of plasmid DNA. Following recovery outgrowth for 1 h, cells were plated onto LB agar medium supplemented with 15 μg/mL kanamycin sulfate. Plates were incubated at 30 °C and colonies that appeared overnight were marked. The largest of these colonies were often found by colony PCR to have integrated plasmid concatemers (or other forms of multiple copy plasmid integrants). Plates were further incubated overnight at room temperature (~24 °C) and moderately sized isolated colonies were patched to LB agar plates and incubated overnight. Colonies were screened by colony PCR to confirm single copy plasmid integration events.

### 2.3 Fluorescent plate reader assays

5 mL LB medium was inoculated from glycerol stocks and incubated overnight at 30 °C, 200 rpm for starter cultures. 96-well plate glycerol stock cultures were prepared by adding 150 μL LB broth to each well in white-walled, μClear® flat-bottom, 96-well plates (Greiner Bio-One) with an optically clear lid. For each assay, wells were inoculated with 1 μL of appropriate overnight culture, and incubated at 30 °C with medium shaking in a Synergy Mx plate reader (BioTek). 50 μL of 50% glycerol was mixed with cultures (final 12.5% glycerol) and plates were either used immediately to inoculate an assay plate or covered with Titer Tops® sealing film and stored at −80 °C. 96-well fluorescence assays were performed with 150 μL LB broth/well in black-walled, μClear® flat-bottom, 96-well plates (Greiner Bio-One) with an optically clear lid. Plates were inoculated from thawed glycerol stock cultures using a sterile 96-pin replicator (Phenix Research Products) – an estimated 1% inoculum. Assay plates were incubated at 30 °C with medium shaking in a Synergy Mx plate reader, with OD_600_ and F_510,530 (85 sensitivity)_ readings taken every 10 min.

mNeonGreen production rate was calculated by measuring rate of change in fluorescence (∆F_510,530_) divided by rate of change in cell density (∆OD_600_) during mid-log growth (OD_600_ values of ~0.15–0.40) using the least squares linear regression equation below:

$$b=\sum_{i=1}^{n}\left(x_{i}-\overset{®}{x}\right)\left(y_{i}-\overset{®}{y}\right)/\sum_{i=1}^{n}{\left(x_{i}-\overset{®}{x}\right)}^{2}$$

in which *b* is the slope of the linear regression best fit line for F_510,530_ versus OD_600_, *x* is the OD_600_ and *y* is the relative fluorescence.

Background fluorescence slightly decreased during growth in strains without the reporter, and was accounted by subtracting the slope of a promoterless reporter strain from the assayed strains. The final equation used is:

$$\mathrm{mNeonGreen}\mathrm{production}=b_{\mathrm{sample}}-b_{\mathrm{promoterless}\mathrm{control}}$$

## 3 Results

### 3.1 Bacteriophage Bxb1 serine integrase allows efficient unidirectional genomic integration of plasmid DNA

To identify serine integrases that can function efficiently in *P. putida* KT2440, we constructed several *P. putida* KT2440 derivative strains (Table 1) expressing serine recombinases and the associated *attB* sequences from the bacteriophages ΦBT1, ΦC1, RV, and BxB1 (Supplemental Fig. 1). The tac promoter (de Boer et al., 1983), which has previously been used for constitutive expression in KT2440 (Johnson and Beckham, 2015), was used for expression of the integrases. These constructs were used to replace *hsdR*, the nuclease component of a putative Type I restriction system. The *hsdR* gene encodes a restriction nuclease, and was chosen as a site for gene replacement due its known inactivation in KT2440 (Bagdasarian and Timmis, 1982), making it a likely neutral site for gene replacement. An *hsdR* deletion strain, without an inserted integrase, was constructed for use as a background control.

The functionality of each serine phage integrase was assayed using a plasmid transformation assay. All five mutant strains, as well as wild-type KT2440, were transformed with equal amounts (by mass) of a replicating vector (pBBR-MCS-2), a suicide vector designed to delete the non-essential gene *PP_0545* (pΔPP_0545) via homologous recombination and *sacB* counter-selection but only serves as a homologous recombination control here, and a suicide plasmid bearing 12 distinct *attP* sequences (pPolyAttP). Transformants with pBBR1-MCS-2 are expected to contain the autonomously replicating plasmid. Transformants with p∆PP_0545 arise from homologous recombination between the genome and 1 kilobase homology arms in the plasmid catalyzed by the endogenous DNA repair machinery. Transformants with pPolyAttP are expected to result from integrase-catalyzed recombination between the *attB* site in the genome and the corresponding *attP* site in the plasmid (Fig. 1A). This integrates the entire plasmid and generates distinct *attL* and *attR* sites flanking the site of integration. Notably, the serine integrase is unable to catalyze recombination between the *attL* and *attR* sites to reverse the plasmid integration.

Transformation efficiency was determined by enumerating transformed colonies selected by 24 h of growth on LB supplemented with 50 μg/mL kanamycin sulfate (Fig. 1B). Transformation efficiency with the replicating vector pBBR1-MCS-2 (Fig. 1B, black bars) was relatively high in all strains, ranging between 6.06×10^4^ (Δ*hsdR*::ΦBT1) and 1.47×10^6^ (Δ*hsdR*::RV) colony forming units (CFU) per microgram of plasmid DNA. Transformation efficiency with the suicide vector pΔPP_0545 (Fig. 1B, white bars), which is designed to integrate into the chromosome via homologous recombination, was relatively inefficient in all strains ranging between 5 (Δ*hsdR*::ΦBT1) and 59 (Δ*hsdR*::RV) CFU/μg of DNA. This was approximately four orders of magnitude lower than pBBR1-MCS-2 in all strains. The similarity in transformation efficiency between wild-type and the Δ*hsdR* strain suggest that deletion of *hsdR* has no effect upon transformation in KT2440, which is to be expected as the restriction enzyme *hsdR* is thought to be inactivated by a mutation in KT2440 (Bagdasarian and Timmis, 1982). Of note, the genomes of strains obtained by single homologous recombination events are not stable and require laborious and time-consuming counter-selection (see Section 2.2.1 of Section 2 for details) to remove the plasmid backbone. Furthermore, in the absence of a selective pressure associated with the desired modification, at most 50% of colonies obtained upon counter-selection will contain the desired mutation, necessitating use of a screening method such as PCR to identify correct strains.

The previous transformation controls give us parameters for high and low efficiency to evaluate the recombination efficiency of each serine integrase in KT2440. As expected the wild-type and Δ*hsdR* strains, which lack an integrase, had zero transformants with pPolyAttP (Fig. 1B, gray bars). Very few pPolyAttP transformants were obtained with Δ*hsdR*::ΦBT1 and Δ*hsdR*::ΦC1 strains, with efficiencies of 2.8 and 0.6 CFU/μg of DNA respectively. However, pPolyAttP transformation efficiency was much higher in the Δ*hsdR*::RV and Δ*hsdR*::BxB1 strains, 2.45×10^3^ and 2.28×10^4^ CFU/μg of DNA respectively, with the BxB1 efficiency approaching that of pBBR1-MCS-2. Integration of pPolyAttP at the *attB* site was assayed by colony PCR in a set of representative transformant colonies for each integrase containing strain (Supplemental Fig. 2). Colony PCR verified plasmid integration at the *attB* loci for all integrases except ΦBT1. We were unable to verify pPolyAttP integration by colony PCR for ΦBT1 colonies, and given its poor performance in the transformation assays we did not pursue further use of this recombinase. Nonetheless, both RV and BxB1 integrases functioned efficiently for plasmid integration in KT2440. However, the Δ*hsdR*::RV strain demonstrated a phenotype in which cells clumped and settled to the bottom of the tube upon reaching stationary phase in liquid cultures. While the mechanism of clumping is unknown, the possibility of genome instability caused by this DNA recombinase raised concerns about its downstream usefulness. As the Δ*hsdR*::BxB1 strain lacked this phenotype, and produced the highest pPolyAttP plasmid integration efficiency, we used this strain for all downstream experiments.

### 3.2 Utilization of BxB1 integrase to rapidly test protein expression in P. putida

**Fig. 2:**
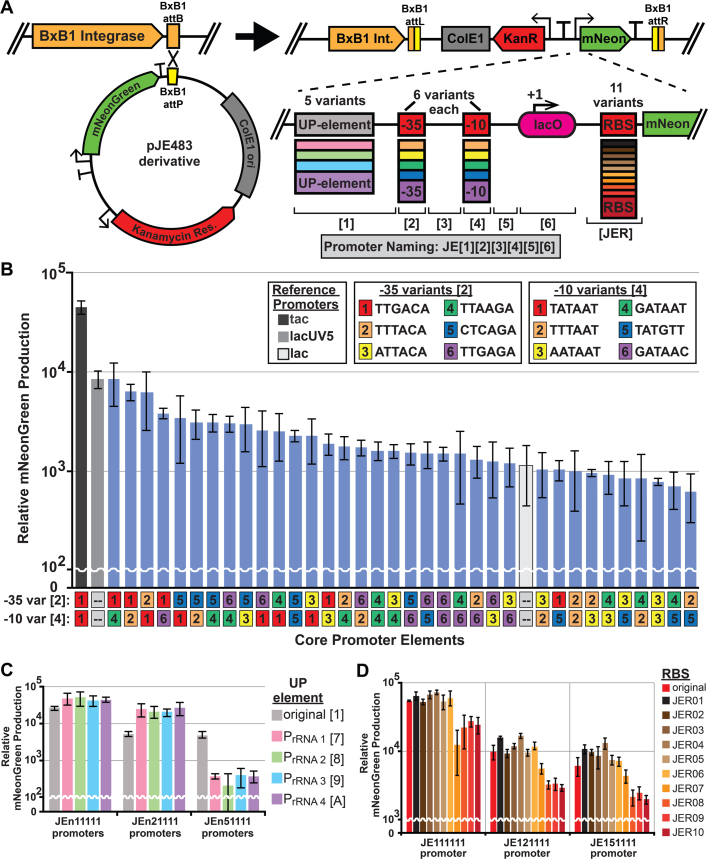
mNeonGreen production assays to determine promoter and ribosomal binding site (RBS) strength in *Pseudomonas putida* KT2440 strain Δ*hsdR*::*BxB1int-attB*. **(A)** Graphic representation of reporter plasmid integration with a diagram of promoter and RBS components used in the study. **(B)** mNeonGreen production by strains harboring mNeonGreen driven by members of the −35 and −10 variant constitutive promoter library. The −35 and −10 variant sequences are indicated above the chart, and particular variants in each promoter indicated beneath the chart. Positions [1, 3, 5], and [6] of the promoter are all variant 1. **(C)** mNeonGreen production by strains harboring one of three −35 variants with positions [3, 4, 5], and [6] of the promoter all variant 1, and one of five position [1] (UP-element) variants. **(D)** mNeonGreen production by strains harboring one of three −35 variants with positions [1, 3, 4, 5], and [6] of the promoter all variant 1, and one of 11 RBS variants each. **(B-D)** All mNeonGreen production values represent the average of three independent experiments, with error bars representing the standard deviation of mNeonGreen production. (For interpretation of the references to color in this figure legend, the reader is referred to the web version of this article).

We used the BxB1 serine integrase to rapidly construct strains containing a promoter library based upon the *tac* promoter. Wild type *P. putida* does not encode the transcription factor LacI, preventing regulation via the *lacO* operator sequence. Specifically, we characterized the rates of mNeonGreen production with 36 constitutive σ^70^ promoters, derived from all combinations of 6 distinct −35 and −10 box sequences (Fig. 2A, Supplemental Table S4), during mid-logarithmic growth. Promoters are named JE[1][2][3][4][5][6] (see Fig. 2A), where values in the [2] and [4] positions represent −35 and −10 variants respectively. Values in the [1, 3, 5], and [6] positions refer to variants of upstream (of −35) sequences, sequences between −35 and −10 elements, downstream (of −10) sequences, and further downstream operator/5′UTR sequences respectively. Sequences used here for −35 and −10 variants were derived from their respective sequences in a small lac promoter variant library used to tune expression of LacZ in *E. coli* (Brewster et al., 2012). Promoters were cloned upstream of an insulated mNeonGreen expression cassette in the plasmid pJE483 (Fig. 2A), which contains a kanamycin resistance cassette and BxB1 *attP* site. Plasmids were transformed into the *P. putida* strain JE90 (KT2440 Δ*hsdR*::BxB1*int*-*attB*), and single copy integration at the BxB1 *attB* site was verified by colony PCR (data not shown). To check for read-through transcription, we compared strains harboring integrated pJE482 (no mNeonGreen gene) and pJE483 (no promoter) plasmids. Fluorescence was not significantly different between the two strains, demonstrating the insulation of the reporter gene (data not shown). Background fluorescence was accounted for by subtracting fluorescence of the promoterless reporter strain from all samples. In addition to the tac promoter (Fig. 2B – dark gray bar), we used the *E. coli* lac (Fig. 2B – white bar) and lacUV5 (Fig. 2B – light gray bar) promoters as references for promoter strength. Unsurprisingly, the tac promoter, which contains consensus −35 and −10 sequences for σ^70^ promoters but non-consensus spacing of 16 bases rather than 17, produced the most mNeonGreen protein. Similar to results in *E. coli*, expression of the reporter with the tac promoter is 5.3-fold higher than with the lacUV5 promoter (de Boer et al., 1983) and 40-fold higher than with the lac promoter. These promoters cover a 72-fold range of expression, with the tac promoter being highest and the JE121511 promoter being lowest (Fig. 2B – blue bars, Supplemental Table S6).

In addition to the −35 and −10 boxes, some promoters contain elements upstream of the −35 box, called UP-elements (Fig. 2A), which can further increase transcription rates from σ^70^ promoters (Ross et al., 1993). UP-elements are typically species-specific and are commonly found in ribosomal RNA promoters. These elements are thought to function by increasing binding affinity for the alpha subunit of RNA polymerase. In an attempt to further increase the range of expression in the library, we incorporated 4 similar, but distinct, UP-elements from the ribosomal RNA promoters in KT2440 upstream of 3 constitutive promoters from the library (Supplemental Table S4). Generally, when one is using an UP-element, it is to increase transcription rates, so we chose three high (JE111111) to medium-high (JE121111 & JE151111) strength promoters to use as a test platform for incorporating UP-elements into synthetic promoters. Incorporation of rRNA promoter UP-elements increased expression from the JE111111 (*tac* promoter) by ~1.7 to 2-fold and JE121111 promoter by ~4 to 5-fold (Fig. 2C, Supplemental Table S7). Unexpectedly, incorporation of the same UP-elements significantly decreased expression from the JE151111 promoter by ~12 to 23-fold.

Protein expression can also be tuned post-transcriptionally by many factors including start codon sequence (O'Donnell and Janssen, 2001), ribosomal binding site sequence and spacing (Salis et al., 2009), RNA secondary structure (Mortimer et al., 2014), and translation inhibiting RNA-protein interactions (Moreno et al., 2007). The original 5′ UTR and RBS sequence used is a derivative of the constitutive expression cassette in pTAC-MAT-Tag-2 (Sigma Aldrich). To further tune protein expression, we generated 10 RBS variants by mutating 4 nucleotides of the core ribosomal binding site (Supplemental Table S5) in plasmids containing promoters JE111111 (tac), JE121111, and JE151111 (Fig. 2D, Supplemental Table S8). The RBS variants utilized with each promoter mostly clustered into two groups, with RBS JER07 being in between, and protein production levels varied by ~6–7-fold from the highest to lowest strength RBS.

### 3.3 Improvement of BxB1 integrase target plasmids

**Fig. 3:**
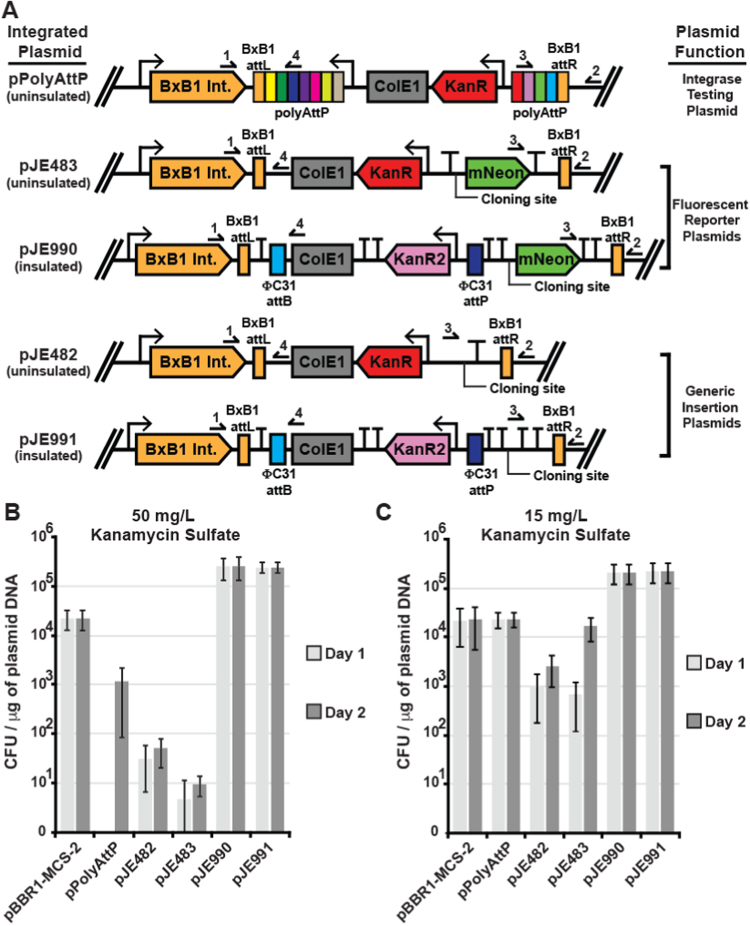
Design and testing of improved integrase target plasmids. **(A)** Graphic representation of integrase-catalyzed site-specific recombination between the BxB1-*attP* (P) of 5 plasmids and the BxB1-*attB* (B) site in the genome of Δ*hsdR*::*BxB1int-attB* using glyphs from the Synthetic Biology Open Language Visual graphical notation. Numbers above primer arrows represent the primers used for screening as described in Supplemental material. Red arrow (KanR) and Pink (KanR2) represent kanamycin resistance markers from pUC57 and pK18mobsacB, respectively, expressed from the original promoters from the source vectors. Green arrow (mNeon) represents mNeonGreen ORF. Directionality of terminators is not indicated, but double terminators are in opposing orientations to insulate elements. **(B & C)** Number of colony forming units observed after 1 (light gray) or 2 (dark gray) days of incubation following transformation of Δ*hsdR*::*BxB1int-attB* with the replicating pBBR1-MCS-2 plasmid or 5 BxB1-*attP* containing plasmids. All error bars represent standard deviation in colony counts from 3 independent transformation experiments. Results are charted for selection on **(B)** 50 mg/L and **(C)** 15 mg/L kanamycin sulfate. (For interpretation of the references to color in this figure legend, the reader is referred to the web version of this article).

During the construction of promoter and RBS testing strains we observed significantly reduced colony formation and increased sensitivity to kanamycin with the reporter plasmid pJE483 when compared to pPolyAttP. Closer examination of the plasmid features and their orientation upon integration revealed that transcription from the opposing BxB1 integrase could potentially suppress expression of the kanamycin selection markers of pJE482 and pJE483 (Fig. 3A) (Brantl, 2007, Shearwin et al., 2005). Transcription from opposing promoters has been demonstrated to form anti-sense RNAs which can reduce translational efficiency or promote mRNA degradation/instability through a variety of mechanisms (Brantl, 2007). Additionally, convergent expression in the absence of terminators has been demonstrated to act through other mechanisms, such as RNA polymerase collisions that lead to the premature termination of the transcriptional progress of both polymerases (Shearwin et al., 2005). With several of the mechanisms described in these two reviews, the repressive effect of opposing Bxb1 transcription on the kanamycin selection marker may be mitigated with the pPolyAttP plasmid, due to the presence of an opposing lac promoter upstream of the kanamycin selection marker (Fig. 3A). Nonetheless, transformants for plasmids with all three backbones were heterogeneous in size and growth rate on selective plates, and larger colonies for pJE482 and pJE483 often had multiple insertions, as determined by a multiplex PCR screen (Supplemental Fig. S3).

We designed a second generation of BxB1 target plasmids to address these issues, generating plasmids pJE990 (reporter plasmid) and pJE991 (generic cloning vector). First, as we could not rule out the possibility that the Kan resistance cassette in pUC57-Kan generally performs poorly at single-copy in *P. putida*, we replaced the pUC57-Kan resistance cassette (Fig. 3A, KanR) with the resistance cassette from pK18mobsacB (Fig. 3A, KanR2), which functions well at single-copy in *P. putida* (Johnson and Beckham, 2015). Second, we insulated the kanamycin resistance gene with upstream and downstream double Rho-independent terminators to reduce possible opposing transcription. Each double terminator is comprised of a strong *E. coli* terminator and a member of a synthetic terminator library (Chen et al., 2013) in opposite orientations. Third, an additional strong Rho-independent *E. coli* terminator from this library was placed downstream of the BxB1 *attP* site to provide a terminator for the upstream BxB1-integrase cassette. Both pJE990 and pJE991 contain ΦC31 *attB* and *attP* sites that could allow excision of the backbone by ΦC31 integrase for reuse of selection marker in the future. Both pJE990 and pJE991, as well as pJE482 and pJE483, contain dual BbsI restriction sites for Golden Gate cloning purposes (Engler et al., 2009).

Plasmid transformation assays were performed to compare transformation efficiency and kanamycin sensitivity of the first generation integrase target plasmids (pPolyAttP, pJE482 & pJE483) with the second generation of integrase target plasmids. The *P. putida* strain JE90 (KT2440 Δ*hsdR*::BxB1) was transformed with equal amounts (by mass) of all 5 of the integrase target plasmids (Fig. 3A), with the replicating plasmid pBBR1-MCS-2 as a control. Kanamycin sensitivity was assayed by incubating equal fractions of each transformation onto LB supplemented with either 50 mg/L (Fig. 3B) or 15 mg/L (Fig. 3C) kanamycin sulfate, henceforth referred to as Kan50 and Kan15, respectively. Growth rate, under selection, was assayed by enumerating colonies after 16 h (1 day) or 40 h (2 days) of growth at 30 °C. With the replicating plasmid (Fig. 3B-C) we obtained similar transformation efficiency on both Kan50 and Kan15 medium, with little change in colony counts from day 1 to day 2. Although colony counts increased from day 1 to day 2 with the first generation *attP* plasmids on Kan50 medium, these counts were still approximately 1 (pPolyAttP) to 3 (pJE483) orders of magnitude lower than the replicating plasmid (Fig. 3B). Transformation efficiency for all three first generation *attP* plasmids is improved by over an order of magnitude by switching to less selective Kan15 medium (Fig. 3C), improving transformation efficiency of pPolyAttP to that of pBBR1-MCS-2. Nonetheless, even on the Kan15 plates, transformants of the first generation of *attP* plasmids displayed significant size heterogeneity, grew poorly in the presence of kanamycin during subsequent passages, and still demonstrated variability in integrated copy number. Multiplex PCR assays demonstrate that 30 out of 30 screened colonies contain the plasmid integrated at the correct locus for the first generation integrase target plasmids pJE482 and pJE483. However, approximately 13% and 10% of colonies obtained with pJE482 and pJE483, respectively, contained multiple integrated plasmids when obtained using Kan15 medium for selection (data not shown). However, the second-generation *attP* plasmids (pJE990/991) are a significant improvement over the first generation. Transformation efficiency with these two plasmids was at least an order of magnitude higher than all of the other plasmids on both Kan50 and Kan15 medium (Fig. 3B–C). Furthermore, their colonies had no size heterogeneity, did not require an additional day of growth, could be readily passaged on Kan50 medium, and each had a single plasmid integrated when 100 colonies were examined (data not shown). Taken together, the second-generation *attP* plasmids are a significant improvement over the initial plasmids.

## 4 Discussion and conclusions

Rapid metabolic engineering of organisms such as *P. putida* will be critical for efforts to create organisms that can add value to the lignin fraction of plant biomass. The BxB1 integrase system developed here for *P. putida* will dramatically increase the speed of the build phase in the iterative design-build-test-learn cycle, allowing constructs to be ready for testing in two days. This approach is substantially faster than using homologous recombination to stably “knock-in” genes into the chromosome, which takes a minimum of five to six days. The unidirectional site-specific recombination also eliminates the instability and copy number issues associated with autonomously replicating plasmids. Further, replicating plasmids are generally unsuitable for industrial use as they require constant selection, which is cost-prohibitive; our integration system should provide more reliable predictive value for chromosomal protein expression because all constructs are at single copy. Additionally, we have routinely integrated 6 kb plasmids with the Bxb1 integration system, each containing a 3.5 kb pathway, without a noticeable drop in transformation efficiency (unpublished data, Elmore & De Capite). We expect that any size limitations for plasmid integration will be determined by the ability to physically transfer large DNA molecules into the cell. While the first generation plasmid designs worked best with the use of lower kanamycin concentrations or longer incubation times, the development of a second generation of plasmids eliminated these drawbacks and will make this toolset easier to use.

The utility of serine integrases has been demonstrated in a broad range of organisms, including mammals (Keravala et al., 2006), plants (Thomson et al., 2012), yeast (Xu and Brown, 2016), archaea (Guss et al., 2008), and bacteria (Siuti et al., 2014). Many other serine integrases are used as genetic tools in other organisms (Brown et al., 2011, Keravala et al., 2006, Xu and Brown, 2016), and many of them may be suitable for expanding the repertoire of genetic tools in *P. putida.* Similar to the CRIM system in *E. coli* (Haldimann and Wanner, 2001a), the development of a temperature-sensitive replicating plasmid for *P. putida* would also expand the utility of serine integrases and serve as a platform for many other recombineering tools. To our knowledge, no temperature sensitive plasmids have yet been developed for *P. putida*, but this advance would add further value to the serine recombinase tools developed here by enabling transient expression during the initial integration as well as unidirectional removal of antibiotic resistance genes in a manner similar to the bidirectional Flp-*frt* and Cre-*lox* systems. This system could also be expanded by using other serine recombinases for which *attB* and *attP* sites have been characterized (Brown et al., 2011).

Many enzymes and pathways require a delicate balance of subunits (Davis et al., 2000) and pathway components for optimal activity (Jones et al., 2015). Here we have begun the development of a suite of genetic elements to tune expression in *P. putida*. For example, the JE711111 promoter (tac + rRNA UP-element) would be suitable for very high expression of rate limiting cytosolic enzymes. A medium-high expression promoter, such as JE131111, could be used for non-rate limiting enzymes to reduce the metabolic load of an exogenous pathway. A medium-low promoter like JE131311 may be more suitable for circumstances where very strong expression could be inappropriate, such as for transcription factors or membrane proteins. Interestingly, not all promoters performed as expected. In particular, variants of JE151111 with the addition of any of the four UP elements significantly reduced rather than enhanced mNeonGreen production. Because of the enigmatic nature of this decrease, we would recommend avoiding these four promoter variants. Ultimately, this library is a starting point, and we expect that additional tuning of RBS, UP, −35 box, and −10 box elements will allow researchers to further broaden the dynamic range for rationally designed protein expression. Furthermore, we expect the knowledge gained from tuning the −35 and −10 box elements in the *tac* promoter will be useful for rationally tuning expression in other σ^70^ promoters of interest, such as those with useful transcription factor operator sequences. The mNeonGreen reporter gene construct will also serve as an ideal testing device for the characterization of other genetic parts, such as terminators or more complex regulatory circuits.

In conclusion, the development of the BxB1 integrase system and promoter library will significantly alter the landscape of what is possible in *P. putida* engineering. The decreasing cost of synthetic DNA (Kosuri and Church, 2014), affordable high-throughput DNA sequencing technologies (Goodwin et al., 2016), automated high-throughput assays, and efficient genome editing technologies has allowed high-throughput engineering efforts to become a reality in model organisms such as *Escherichia coli* and *Saccharomyces cerevisiae*. With the genome editing technology developed here, *P. putida* is much closer to fully exploiting the same technological advances.

## Declarations of Interest

We wish to confirm that there are no known conflicts of interest associated with this publication and there has been no significant financial support for this work that could have influenced its outcome.
