# Supplementary material for: Development of a high efficiency integration system and promoter library for rapid modification of Pseudomonas putida KT2440
Source: Metab Eng Commun. 2017 Apr 15;5:1–8. doi: 10.1016/j.meteno.2017.04.001 (PMC5699527; doi:10.1016/j.meteno.2017.04.001)
Supplement: Supplementary file 2 — Supplementary material [file mmc2.docx]

**Supplemental Figure 1**

**Supplemental Figure 1.** Graphical diagram of genome arrangement at the *hsdR* loci for strains assayed in Figure 1 using glyphs from Synthetic Biology Open Language (SBOL) Visual conventions.

**Supplemental Figure 2**

**
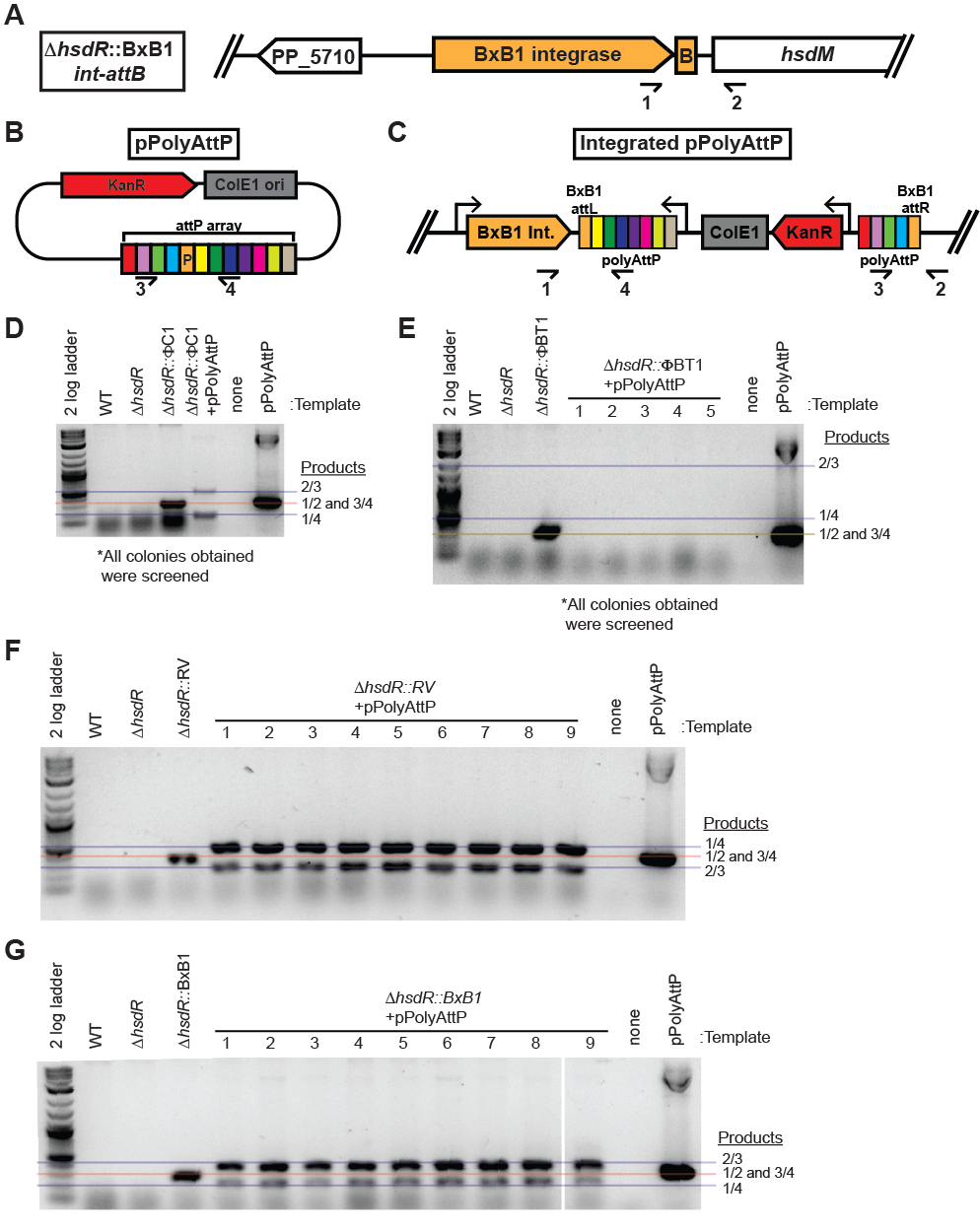
**

**Supplemental Figure 2.** Colony PCR validation of pPolyAttP integration. **(A-C)** SBOL visual diagram of the *hsd* locus of Δ*hsdR*::*BxB1int-attB* **(A)**, pPolyAttP **(B)**, and pPolyAttP integrated into Δ*hsdR*::*BxB1int-attB* **(C)** with primer binding locations indicated. **(D-G)** Gel electrophothesis analysis of colony PCR results to validate pPolyAttP integration into the ΦC1 **(D)**, ΦBT1 **(E)**, RV **(F)** and BxB1 **(G)** attB sites of the respective Δ*hsdR*::*int-attB* sites. Product sizes expected for primer combinations (left) in parent strain (orange lines), naked plasmid (orange lines) and for integrated plasmid strains (blue lines) are indicated. Note, screening for all transformant colonies obtained with integrases ΦC1 **(D)** & ΦBT1 **(E)** is shown. Screening for nine representative transformant colonies obtained with integrases RV **(F)** and BxB1 **(G)** are shown with the primer binding locations indicated with arrows labeled 1-4.

**Supplemental Figure 3**

**
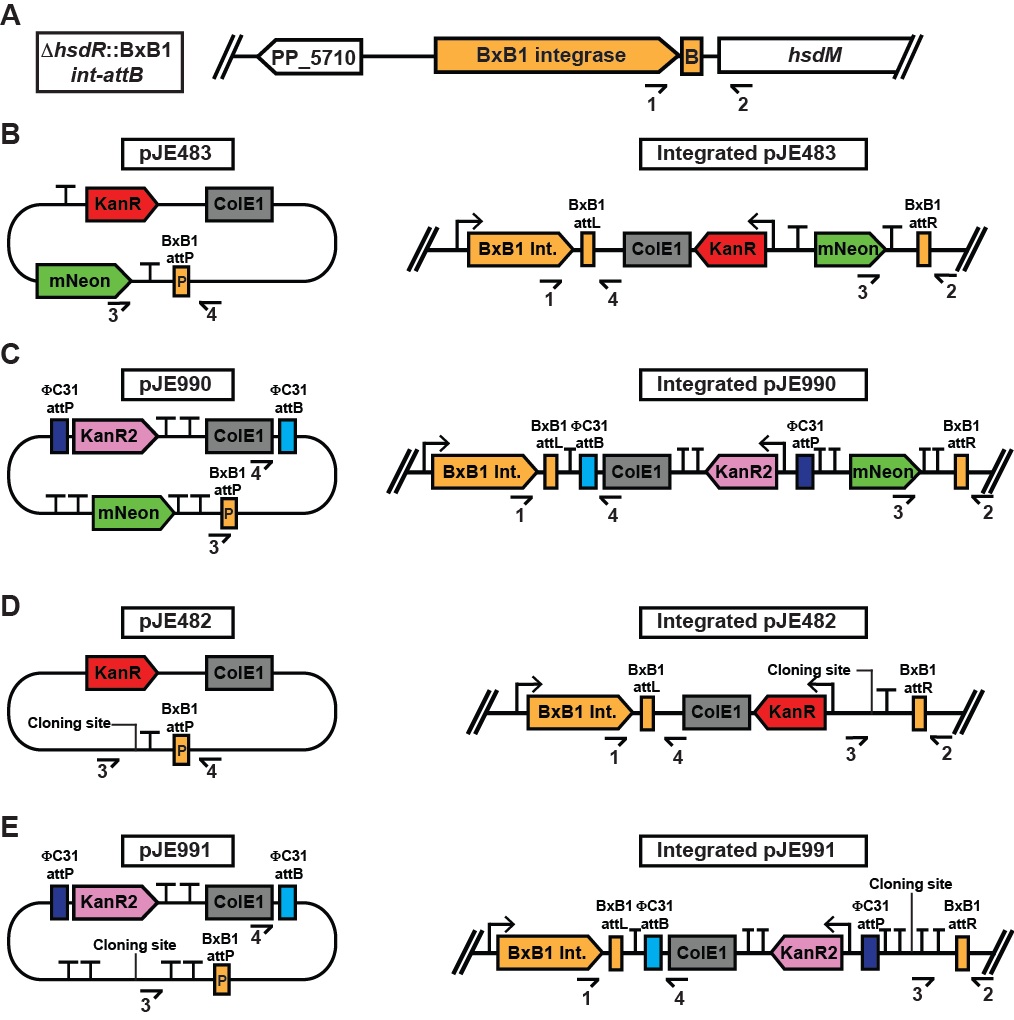
**

**Supplemental Figure 3.** Multiplex PCR primers for screening plasmid integration into the Bxb1 attB site. **(A)** Primer binding locations for primers 1/2 in the parental strain (JE90) genome. **(B-E)** Primer binding locations in both the free plasmid (primers 3/4 - unique to each plasmid) and integrated plasmids (primers 1/2/3/4) for plasmids pJE483 **(A)**, pJE990 **(B)**, pJE482 **(B)**, and pJE991 **(E)**. Primer sequences are listed in Supplemental Table S4.

**Supplemental Methods**

**Plasmid Construction**

Phusion® High Fidelity Polymerase (Thermo Scientific) and primers synthesized by Integrated DNA Technologies (IDT) or Eurofins Genomics were used in all PCR amplification for plasmid construction. Plasmids were constructed using NEBuilder® HiFi DNA Assembly Master Mix (New England Biolabs - NEB) or T4 DNA ligase (NEB) according to manufacturer’s instructions. Plasmids were transformed into either competent Top10 (Life Technologies), NEB 5-alpha F’I^q^ (NEB), or Epi400 (Epicentre Biotechnologies) *Escherichia coli* according to manufacturer’s instructions. Transformants were selected on LB (Miller) agar plates containing 50 mg/L kanamycin sulfate for selection and incubated at 37 °C. Sequences were confirmed using Sanger Sequencing performed by GenScript or Eurofins Genomics.

Inserts for plasmids pPolyAttP, pΔPP_4740, and all pΔPP_4740 derivatives containing a phage integrase and attB sequence were synthesized by GenScript. The phage integrase genes were codon optimized by GenScript. The inserts for pΔPP_4740 derivatives were subcloned into the EcoRI / HindIII sites of pK18mobsacB. The insert for pPolyAttP was inserted into the NotI site of pUC57.

Plasmid p∆PP_0545 was constructed by amplification of 1kb upstream and downstream of PP_0545 using primers PP_0545_UP_fwd/rev and PP_0545_DN_fwd/rev respectively, and assembled into the HindIII site of pK18mobsacB.

Plasmid pJE432 was constructed by amplification of the pUC57-Kan backbone in pPolyAttP using using primers (oJE186/187) called pJE432-435_bb_F/R. The insert for pJE432 was amplified from gBlock called pJE432_insert. Primers for insert amplification are pJE432-435_ins_F/R (oJE188/189). Overhangs for assembly are included in gBlock insert. Plasmid pJE481 was constructed by linearizing pJE432 with XhoI/NotI, and ligation with annealed 5’-phosphorylated oJE263/264. Plasmid pJE482 was constructed by linearizing pJE481 with XhoI/BamHI, and ligation with annealed 5’-phosphorylated oJE266/267.

Plasmid pJE433 was constructed by amplification of pUC57-Kan backbone in pPolyAttP, using primers (oJE186/187) called pJE432-435_bb_F/R. Primer pair oJE188/192 was used for amplification of pJE434-ins-UP gBlock, and primer pair oJE189/193 for pJE434-ins-DN gBlock. Overhangs for assembly are included in gBlock inserts. Plasmid pJE479 was constructed by linearizing pJE434 with BsaI, and ligation with annealed 5’-phosphorylated oligos oJE260/261. Plasmid pJE483 was constructed by linearizing pJE479 with NotI/XbaI and ligation with NotI/XbaI digested pJE483_484_ins gBlock.

Plasmid pJE990 was constructed by assembly of 4 fragments. The fragments were generated by amplification of the pUC origin from pK18mobsacB, and gBlocks pJE990_gBlock_1/2/3 with oligos oJE520/521, oJE522/523, oJE524/525, and oJE526/527 respectively. Plasmid pJE991 was constructed by assembly of 3 fragments. The fragments were generated by amplification of the pUC origin from pK18mobsacB, pJE990_gBlock_1, and pJE991_gBlock_2 with oligos oJE520/521, oJE522/523, oJE524/527 respectively.

Plasmids pJE541- pJE576 & pJE943-954 were constructed by ligating BbsI-linearized pJE483 with two sets of annealed 5’-phosphorylated oligos for promoter construction. Promoters were constructed using 5’- oligo pairs oJE-UP##_+/- and oJE-DN****_+/- to construct promoter JE##****.

Plasmids pJE955 to pJE957 were constructed by quickchange PCR mutagenesis with primers oJE489/490. Plasmids pJE958-967, pJE968-977, and pJE978-987 were constructed by linearizing pJE955, pJE956, and pJE957 respectively with AflII / NdeI and ligation with 5’-phosphorylated annealed oligos JER##_+/- to construct RBS JER##.

**5’-Phosphorylation and annealing of oligo pairs**

Oligo pairs were simultaneously 5’-phosphorylated and annealed in a single reaction. Reactions contained 10 μM of each oligo, 50 mM Tris-HCl, 10 mM MgCl2, 1 mM ATP, 10 mM DTT, 5 U T4 polynucleotide kinase @ pH 7.4. Combined phosphorylation and annealing reactions were incubated at 37 °C for 30 minutes, denatured at 95 °C for 5 minutes, and annealed by cooling to 25 C at a rate of 2 °C/min.

**Colony PCR verification of integrated plasmids**

Colony PCR was utilized to verify the integration of plasmids at the correct attB locus. For pPolyAttP integration screening primers 1-4 (indicated in Figure 3A and Supplemental Figure 2A-C) are listed below:

***ΔhsdR::ΦBT1int-attB***

| **Primer #** | **Name** | **Sequence** |
| --- | --- | --- |
| 1 | (oJE785) phiBT1_int_L | CACCCGGAGACCAAGAAATA |
| 2 | (oJE516) UNIV_attB_R | ACGCCTTCCTGAATCTTGTT |
| 3 | (oJE786) A118_poly_ver_Lv2 | TATCTCACAGGTCCACGGTT |
| 4 | (oJE787) A118_poly_ver_Rv1 | GCAATTAAGCGAGTTGGAAATG |

***ΔhsdR::ΦC1int-attB***

| **Primer #** | **Name** | **Sequence** |
| --- | --- | --- |
| 1 | (oJE788) phiC1_int_L | AGCTGTTTGACGATGAGGTT |
| 2 | (oJE516) UNIV_attB_R | ACGCCTTCCTGAATCTTGTT |
| 3 | (oJE786) A118_poly_ver_Lv2 | TATCTCACAGGTCCACGGTT |
| 4 | (oJE787) A118_poly_ver_Rv1 | GCAATTAAGCGAGTTGGAAATG |

***ΔhsdR::RVint-attB***

| **Primer #** | **Name** | **Sequence** |
| --- | --- | --- |
| 1 | (oJE789) RV1_int_L | GGTAAAAGCGGCCGTATTTT |
| 2 | (oJE516) UNIV_attB_R | ACGCCTTCCTGAATCTTGTT |
| 3 | (oJE786) A118_poly_ver_Lv2 | TATCTCACAGGTCCACGGTT |
| 4 | (oJE787) A118_poly_ver_Rv1 | GCAATTAAGCGAGTTGGAAATG |

***ΔhsdR::BxB1int-attB***

| **Primer #** | **Name** | **Sequence** |
| --- | --- | --- |
| 1 | (oJE790) Bxb1_int_L | ATTTTGGTGACCTGCAAGAG |
| 2 | (oJE516) UNIV_attB_R | ACGCCTTCCTGAATCTTGTT |
| 3 | (oJE786) A118_poly_ver_Lv2 | TATCTCACAGGTCCACGGTT |
| 4 | (oJE787) A118_poly_ver_Rv1 | GCAATTAAGCGAGTTGGAAATG |

For pJE482 (and derivatives) integration screening primers 1-4 (indicated in Figure 3A and Supplemental Figure 3) are listed below:

| **Primer #** | **Name** | **Sequence** |
| --- | --- | --- |
| 1 | (oJE65)_oJE65 | cgacgatagtggcagcatg |
| 2 | (oJE66)_oJE66 | catgtagttgtaggcgtcttc |
| 3 | (oJE1112)_pJE482_intscr_L1 | gagacggtcacagcttgtct |
| 4 | (oJE1131)_pJE482_intscr_R3 | taggcaccccaggcTCTA |

For pJE483 (and derivatives) integration screening primers 1-4 (indicated in Figure 3A and Supplemental Figure 3) are listed below:

| **Primer #** | **Name** | **Sequence** |
| --- | --- | --- |
| 1 | (oJE65)_oJE65 | cgacgatagtggcagcatg |
| 2 | (oJE66)_oJE66 | catgtagttgtaggcgtcttc |
| 3 | (oJE1116)_pJE483_intscr_L1 | TTCAAAGAGTGGCAGAAAGC |
| 4 | (oJE1132)_pJE483_intscr_R3 | taggcaccccaggcGCTA |

For pJE990 (and derivatives) integration screening primers 1-4 (indicated in Figure 3A and Supplemental Figure 3) are listed below:

| **Primer #** | **Name** | **Sequence** |
| --- | --- | --- |
| 1 | (oJE65)_oJE65 | cgacgatagtggcagcatg |
| 2 | (oJE66)_oJE66 | catgtagttgtaggcgtcttc |
| 3 | (oJE1120)_pJE990_intscr_L2 | ATGTCATGGGTATGGACGAA |
| 4 | (oJE1121)_pJE990_intscr_R2 | cacctagatccttttggagtacg |

For pJE991 (and derivatives) integration screening primers 1-4 (indicated in Figure 3A and Supplemental Figure 3) are listed below:

| **Primer #** | **Name** | **Sequence** |
| --- | --- | --- |
| 1 | (oJE65)_oJE65 | cgacgatagtggcagcatg |
| 2 | (oJE66)_oJE66 | catgtagttgtaggcgtcttc |
| 3 | (oJE1123)_pJE991_intscr_L2 | cgataattcaGGTagaagacaaCTGG |
| 4 | (oJE1121)_pJE990_intscr_R2 | cacctagatccttttggagtacg |

**Synthesized DNAs used in this study**

**1. Fragments synthesized by GenScript**

∆PP_4740 DNA fragment (subcloned into EcoRI/HindIII site of pK18mobsacB)

gcatttacgagctcatttcttgccttgggtctgtaagcgtcgctaagccgtatgacgcagtcctgggtcttcaaaataatttcagcacgctcctttgagcccagctctttggaatccatcatcgttgcaagttgggtactcaacgagtccttcatcgatgcactgaaatctacccctttagacttggtcgcatccgcatactgaaaagcgcctttgaatgcggcggttacatctgccgaatacccaaggccacatgccttaatcgcctgatcaactggtgcaacagatcgatagggagaaaggcctgaacaagctgacaagaacacagcggaaagcaaccagatatgactacgcattaacaatttctccacccccaaataatggcactaagccactataccacttattcgacgtataggtttggccagagtggcaggcgccacttgcgagcatccacccaacgcgaaaatgtcattaatagtagggctgagctaactgagcgctgacagctgggaacaccttgggcatgcggcttggcttcatgaacaccgatgagcttggtattgcaacgtactggccatgcggctgctgaatcatcatttagaaaagccgtgaggtttccactgccatcagcgcataggaaggcccgacgaccgggcctgagagccccccgacgatagtggcagcatgcctctagctattttgttaaattcggcaacacgccatagggacatagccagcagagctgttgacaattaatcatcggctcgtataatgtgtggaattgtgagcggataacaatttcacacataggaggtgatgcatcacgcatccatgggatccaacctgttcccgcagctgcagaacctgcacgaatacagcaacacaccctcggcctttgtggtgcgcagcgtgtttgaagacgcctacaactacatgaaatccggccagctgctgcgccaggtgatcaacaagattcaggaaggcgtggacttcaacagggcccaggaacgccacgagttcggcaacctctatgaacaattgctgcgcgacctgcagaacgccggcaacgccggtgagttctacacacctcgaccagtcaccgaatttatggtgcgcatggttgatcccaagctggctgaaaaggtcatggacccggcctgcggcaccggcggctttctcacctgcgccatcgagcacaagcgcagacgctatgtaaaaaccgccgaagacgaacgcaccttgcaggccagcatttttggcgtggagaaaaaaccgctgccgcacctgctggccaccaccaacatgatcctgcatggcatcgaagtgcccagccagatccgtcacgacaacaccctgagcaaaccgctgatcagctggggcccaagcgagcgcgtgcattgtatcgtcgccaacccgccgttcggcggcatggaagaagacggtatcgaaacaaattttcctgccgctttccgcacccgggaaaccgccgatttgttcttggtattgattatgcagctgctcaaagatggtggccgcgccgcagtggtactgcccgatggcttcctttttggcgaaggcatcaaaagcc

BxB1 integrase & attB (subcloned into BamHI site of ∆PP_4740 fragment)

TGCAGATGCGTGCGCTGGTTGTGATCCGTCTGAGCCGTGTTACCGATGCGACCACCAGCCCGGAGCGTCAGCTGGAAAGCTGCCAGCAACTGTGCGCGCAACGTGGCTGGGATGTGGTTGGTGTGGCGGAGGATCTGGATGTGAGCGGTGCGGTTGATCCGTTCGACCGTAAACGTCGTCCGAACCTGGCGCGTTGGCTGGCGTTCGAGGAACAGCCGTTTGATGTGATCGTTGCGTACCGTGTTGACCGTCTGACCCGTAGCATTCGTCACCTGCAACAACTGGTGCACTGGGCGGAGGACCACAAGAAACTGGTGGTTAGCGCGACCGAAGCGCACTTCGATACCACCACCCCGTTTGCGGCGGTGGTTATCGCGCTGATGGGTACCGTTGCGCAAATGGAGCTGGAAGCGATCAAGGAACGTAACCGTAGCGCGGCGCACTTCAACATTCGTGCGGGCAAATACCGTGGTAGCCTGCCGCCGTGGGGCTATCTGCCGACCCGTGTTGATGGTGAATGGCGTCTGGTGCCGGACCCGGTTCAGCGTGAGCGTATTCTGGAAGTGTACCACCGTGTGGTTGATAACCACGAACCGCTGCACCTGGTTGCGCACGACCTGAACCGTCGTGGCGTGCTGAGCCCGAAAGATTATTTTGCGCAGCTGCAAGGCCGTGAGCCGCAAGGTCGTGAATGGAGCGCGACCGCGCTGAAGCGTAGCATGATCAGCGAGGCGATGCTGGGCTATGCGACCCTGAACGGTAAAACCGTTCGTGACGATGATGGTGCGCCGCTGGTGCGTGCGGAGCCGATTCTGACCCGTGAGCAGCTGGAAGCGCTGCGTGCGGAACTGGTTAAGACCAGCCGTGCGAAGCCGGCGGTGTCTACCCCGAGCCTGCTGCTGCGTGTTCTGTTCTGCGCGGTTTGCGGTGAACCGGCGTACAAGTTTGCGGGTGGCGGTCGTAAACACCCGCGTTATCGTTGCCGTAGCATGGGTTTCCCGAAGCACTGCGGCAACGGTACCGTTGCGATGGCGGAATGGGACGCGTTTTGCGAGGAACAAGTGCTGGATCTGCTGGGTGATGCGGAGCGTCTGGAAAAAGTGTGGGTTGCGGGTAGCGACAGCGCGGTGGAGCTGGCGGAAGTTAACGCGGAGCTGGTTGACCTGACCAGCCTGATTGGCAGCCCGGCGTATCGTGCGGGTAGCCCGCAGCGTGAAGCGCTGGATGCGCGTATTGCGGCGCTGGCGGCGCGTCAAGAGGAACTGGAGGGTCTGGAAGCGCGTCCGAGCGGTTGGGAGTGGCGTGAAACCGGCCAGCGTTTTGGTGATTGGTGGCGTGAGCAAGACACCGCGGCGAAGAACACCTGGCTGCGTAGCATGAACGTTCGTCTGACCTTCGATGTGCGTGGCGGTCTGACCCGTACCATTGATTTTGGTGACCTGCAAGAGTATGAACAACACCTGCGTCTGGGCAGCGTGGTTGAACGTCTGCACACCGGTATGAGCTAAGCTAGCTCGGCCGGCTTGTCGACGACGGCGGTCTCCGTCGTCAGGATCATCCGGGCGGATCC

RV integrase & attB (subcloned into BamHI site of ∆PP_4740 fragment)

TGCAGATGCGTTACACCACCCCGGTTCGTGCGGCGGTGTATCTGCGTATCAGCGAGGACCGTAGCGGTGAACAGCTGGGCGTTGCGCGTCAACGTGAGGATTGCCTGAAGCTGTGCGGTCAGCGTAAATGGGTTCCGGTGGAATACCTGGACAACGATGTTAGCGCGAGCACCGGCAAGCGTCGTCCGGCGTATGAGCAAATGCTGGCGGACATCACCGCGGGCAAAATTGCGGCGGTGGTTGCGTGGGACCTGGATCGTCTGCACCGTCGTCCGATTGAGCTGGAAGCGTTTATGAGCCTGGCGGATGAAAAGCGTCTGGCGCTGGCGACCGTTGCGGGTGATGTGGATCTGGCGACCCCGCAGGGTCGTCTGGTTGCGCGTCTGAAAGGCAGCGTGGCGGCGCATGAGACCGAACACAAGAAAGCGCGTCAACGTCGTGCGGCGCGTCAAAAGGCGGAGCGTGGTCACCCGAACTGGAGCAAAGCGTTCGGCTACCTGCCGGGTCCGAACGGTCCGGAACCGGACCCGCGTACCGCGCCGCTGGTTAAGCAGGCGTATGCGGATATCCTGGCGGGTGCGAGCCTGGGTGATGTGTGCCGTCAATGGAACGATGCGGGTGCGTTCACCATTACCGGTCGTCCGTGGACCACCACCACCCTGAGCAAGTTTCTGCGTAAACCGCGTAACGCGGGTCTGCGTGCGTACAAGGGTGCGCGTTATGGCCCGGTTGACCGTGATGCGATCGTGGGCAAAGCGCAATGGAGCCCGCTGGTTGACGAGGCGACCTTTTGGGCGGCGCAAGCGGTTCTGGATGCGCCGGGTCGTGCGCCGGGTCGTAAGAGCGTTCGTCGTCACCTGCTGACCGGTCTGGCGGGTTGCGGTAAATGCGGTAACCATCTGGCGGGCAGCTACCGTACCGACGGTCAGGTGGTTTATGTTTGCAAGGCGTGCCATGGTGTGGCGATCCTGGCGGATAACATCGAGCCGATTCTGTACCACATTGTTGCGGAACGTCTGGCGATGCCGGATGCGGTGGATCTGCTGCGTCGTGAGATCCATGATGCGGCGGAGGCGGAAACCATTCGTCTGGAGCTGGAAACCCTGTATGGTGAACTGGATCGTCTGGCGGTGGAGCGTGCGGAAGGTCTGCTGACCGCGCGTCAGGTTAAGATCAGCACCGACATTGTGAACGCGAAGATCACCAAACTGCAAGCGCGTCAGCAAGACCAAGAGCGTCTGCGTGTTTTCGATGGTATCCCGCTGGGTACCCCGCAAGTGGCGGGTATGATTGCGGAGCTGAGCCCGGACCGTTTTCGTGCGGTTCTGGATGTGCTGGCGGAAGTGGTTGTGCAGCCGGTGGGTAAAAGCGGCCGTATTTTCAACCCGGAACGTGTTCAAGTGAACTGGCGTTAAGCTAGCTCTCGTGGTGGTGGAAGGTGTTGGTGCGGGGTTGGCCGTGGTCGAGGTGGGGTGGTGGTAGCCATTCGGGATCC

phiBT1 integrase & attB (subcloned into BamHI site of ∆PP_4740 fragment)

TGCAGATGAGCCCGTTCATTGCGCCGGATGTGCCGGAGCACCTGCTGGACACCGTGCGTGTTTTTCTGTACGCGCGTCAGAGCAAAGGTCGTAGCGATGGCAGCGATGTGAGCACCGAGGCGCAGCTGGCGGCGGGTCGTGCGCTGGTTGCGAGCCGTAACGCGCAAGGTGGCGCGCGTTGGGTGGTTGCGGGTGAGTTCGTGGATGTTGGTCGTAGCGGTTGGGACCCGAACGTGACCCGTGCGGATTTTGAGCGTATGATGGGTGAAGTTCGTGCGGGCGAGGGCGACGTGGTTGTGGTTAACGAGCTGAGCCGTCTGACCCGTAAGGGTGCGCACGATGCGCTGGAGATCGACAACGAACTGAAGAAACACGGCGTGCGTTTCATGAGCGTTCTGGAACCGTTTCTGGACACCAGCACCCCGATCGGTGTGGCGATTTTCGCGCTGATCGCGGCGCTGGCGAAGCAAGACAGCGATCTGAAAGCGGAGCGTCTGAAGGGTGCGAAAGATGAAATTGCGGCGCTGGGTGGCGTTCACAGCAGCAGCGCGCCGTTTGGTATGCGTGCGGTGCGTAAGAAAGTTGACAACCTGGTGATCAGCGTTCTGGAGCCGGACGAAGATAACCCGGATCACGTGGAGCTGGTTGAACGTATGGCGAAAATGAGCTTCGAGGGTGTTAGCGACAACGCGATTGCGACCACCTTTGAGAAGGAAAAAATTCCGAGCCCGGGTATGGCGGAACGTCGTGCGACCGAGAAGCGTCTGGCGAGCGTGAAAGCGCGTCGTCTGAACGGTGCGGAGAAGCCGATCATGTGGCGTGCGCAGACCGTTCGTTGGATTCTGAACCACCCGGCGATCGGTGGCTTCGCGTTTGAACGTGTGAAGCACGGCAAAGCGCACATCAACGTTATTCGTCGTGACCCGGGTGGCAAACCGCTGACCCCGCACACCGGTATTCTGAGCGGCAGCAAGTGGCTGGAGCTGCAAGAAAAGCGTAGCGGTAAAAACCTGAGCGATCGTAAACCGGGTGCGGAAGTGGAACCGACCCTGCTGAGCGGTTGGCGTTTCCTGGGCTGCCGTATCTGCGGTGGCAGCATGGGTCAGAGCCAAGGTGGCCGTAAGCGTAACGGTGACCTGGCGGAAGGCAACTACATGTGCGCGAACCCGAAAGGCCACGGTGGCCTGAGCGTTAAGCGTAGCGAGCTGGATGAGTTCGTGGCGAGCAAAGTTTGGGCGCGTCTGCGTACCGCGGACATGGAGGATGAACACGATCAAGCGTGGATCGCGGCGGCGGCGGAGCGTTTTGCGCTGCAACATGATCTGGCGGGTGTGGCGGATGAGCGTCGTGAACAGCAAGCGCACCTGGACAACGTTCGTCGTAGCATCAAAGACCTGCAAGCGGACCGTAAGCCGGGTCTGTATGTGGGCCGTGAGGAACTGGAAACCTGGCGTAGCACCGTTCTGCAATACCGTAGCTATGAGGCGGAATGCACCACCCGTCTGGCGGAGCTGGATGAAAAGATGAACGGTAGCACCCGTGTGCCGAGCGAATGGTTTAGCGGTGAGGACCCGACCGCGGAAGGTGGCATTTGGGCGAGCTGGGATGTTTACGAGCGTCGTGAATTTCTGAGCTTCTTTCTGGATAGCGTGATGGTTGACCGTGGTCGTCACCCGGAGACCAAGAAATATATCCCGCTGAAGGACCGTGTGACCCTGAAATGGGCGGAACTGCTGAAGGAAGAGGACGAGGCGAGCGAAGCGACCGAGCGTGAACTGGCGGCGCTGTAAGCTAGCGTCCTTGACCAGGTTTTTGACGAAAGTGATCCAGATGATCCAGCTCCACACCCCGAACGCGGATCC

phiC1 integrase & attB (subcloned into BamHI site of ∆PP_4740 fragment)

TGCAGATGAAACGTGCGGCGCTGTACATCCGTGTGAGCACCATGGAACAGGCGAAAGAGGGTTATAGCATTCCGGCGCAAACCGACAAGCTGAAAGCGTTCGCGAAGGCGAAAGACATGGCGGTGGCGAAAGTTTACACCGATCCGGGTTTTAGCGGTGCGAAGATGGAACGTCCGGCGCTGCAAGAGATGATCAGCGACATTCAAAACAAGAAAATCGATGTGGTTCTGGTGTACAAACTGGATCGTCTGAGCCGTAGCCAGAAGAACACCCTGTATCTGATCGAAGACGTGTTCCTGAAGAACAACGTTGATTTCATCAGCATGCAGGAGAGCTTCGACACCAGCACCCCGTTTGGCCGTGCGACCATCGGTATGCTGAGCGTTTTCGCGCAGCTGGAACGTGATACCATTACCGAGCGTATGCACATGGGCCGTACCGAACGTGCGAAGCAAGGTTACTATCACGGTAGCGGCATCGTGCCGCTGGGCTACGACTATGTTCACGGCGAGCTGATCATTAACGATTACGAAGCGCAGATCATTCAAGAGATCTACGACCTGTATGTGAACCAGGGTAAAGGCCAGCAATACATTACCAAACGTATGGTTGCGAAGTATCCGGATAAGGTGAAAACCCTGACCATCGTTAAGTACGCGCTGACCAACCCGCTGTATATCGGTAAAATTAGCTGGGACGGCAAGGTGTACGATGGTCACCACAGCCCGATCATTGACAAAAGCATGTATGATAAGGCGCAGGAAATCATTGCGCGTATGGCGCAAAAAGGTGGCGAGCAGCACGGTAACCAACTGGGCCTGCTGCTGGGTATCACCTACTGCGGCAAGTGCGGCGCGGAAGTGTTTCGTTATGTTAGCGGTGGCAAGAAATACCGTTATAACTACTATATGTGCCGTAGCGTGAAGAAAATGCTGCCGAGCCTGGTTAAAGACTGGAACTGCAAGCAGCCGAGCCTGCGTCAAGAGGTGGTTGAAAAGAAAGTGATCGACAGCCTGAAAAGCCTGGATTTCAAGAAAATTGAGCGTGAACTGAAGCAGGTTGAAAACAAGACCAAAAGCAAGATCACCACCATTAACAACCAAATCAGCAAGAAACACAACGAGAAACAGAAGATTCTGGACCTGTACCAATATGGCACCTTTGATGTTACCATGCTGAACGAACGTATGAAGAAAATCGACAACGAGATTAACGCGCTGACCGCGAACATCGCGAACCTGGAAGGTACCAAAAGCGAGAGCCTGATTAACAAACTGGAAACCCTGAAGACCTTCAACTGGGAGACCGAAACCACCGAGAACAAAATCCTGATCATTAAGGAGTTCGTGGAACGTATTGAGCTGTTTGACGATGAGGTTATCATTAAATACAAGTTTTAAGCTAGCATATTTAACCGCTTCCCGAAAAATTTCGCGTGGATGAGCAATACTTTGATTCAGTGAACCTTTGAAAATCGTTTTCTGTTGGATAAGGATCC

PolyAttP (subcloned into NotI site of pUC57-Kan)

ggtgctgggttgttgtctctggacagtgatccatgggaaactactcagcaccaccaatgttccgcacaggtgtagtgtatctcacaggtccacggttggccgtggactgctgaagaacattccacgccaggatcaaccccgttccagcccaacagtgttagtctttgctcttacccagttgggcgggatagcctgcccgttgtttagttcctcgttttctctcgttggaagaagaagaaacgagaaactaaaattagtcgtggtttgtctggtcaaccaccgcggtctcagtggtgtacggtacaaaccccgacattaaatataaattttagtacatagtgttatatacactaataaacaaaatcatatacctaaaatattacatttccaactcgcttaattgcgagtttttatttcgtttatttcaattaaggtaactaaaaaactccttttaaaaaaatacagcgtttttcatgtacaactatactagttgtagtgcctaaacaaaataaaaaacattgatttttattaacttcttttgtgcggaactacgaacagttcattaatacgaagtgtacaaacttccatacaaaaataaccacgacaattaagacgtggtttctatagttttaaagttggttattagttactgtgatatttatcacggtacccaataaccaatgaatatttgaaggcatgttccccaaagcgataccacttgaagcagtggtactgcttgtgggtacactctgcgggtgatgagagaatactgttgaacaatgaaaaactaggcatgtagaagttgtttgtgcactaactttaa

**2. gBlocks synthesized by Integrated DNA Technologies**

pJE432_insert

**gcgcgtcagcgggtgttggcgggtgtcggggctggcttaaGGATCCatcgatGCTAGCgaattcGGTACCctgcagGACGTCaagcttCTCGAGgcacaggtgtagtgtatctcacaggtccacggttggccgtggactgctgaagaacattccacgccaggagcggccgcagtcaaaagcctccggtcggaggcttttgactagccaaaacaaactaaagcgcccttgtggcgctttagttttTCTAGAgcctggggtgcctaatgagtgagctaactcacattaattg**

pJE434-ins-DN gBlock

GGCATCGCTGACGGTCAACTACCGTTATACCTACGAAGGTAGCCATATTAAGGGCGAAGCTCAAGTGAAGGGTACCGGCTTTCCGGCGGACGGTCCGGTGATGACGAACTCCCTCACCGCCGCCGATTGGTGTCGTAGCAAGAAAACCTATCCGAATGACAAAACCATCATCTCGACGTTTAAGTGGAGCTATACGACGGGTAATGGCAAGCGCTACCGTTCGACGGCACGTACCACCTACACGTTTGCAAAGCCTATGGCTGCAAATTACCTCAAGAATCAACCTATGTATGTGTTCCGGAAAACGGAACTCAAACATAGCAAAACGGAACTGAACTTCAAAGAGTGGCAGAAAGCTTTCACGGATGTCATGGGTATGGACGAACTCTATAAATAAtctagagacgaacaataaggcctccctaacggggggccttttttattgataacaaaagcacaggtgtagtgtatctcacaggtccacggttggccgtggactgctgaagaacattccacgccaggagcggccgcaaaacaaactaaagcgcccttgtggcgctttagttttGCTAGCgcctggggtgcctaatgagtgagctaactcacattaattg

pJE434-ins-UP gBlock

gcgcgtcagcgggtgttggcgggtgtcggggctggcttaaggatccagtcaaaagcctccggtcggaggcttttgactagcacctcggtaccaaattccagaaaagaggcctcccgaaaggggggccttttttcgttttggtccactagtagtcttgagaccaagcAATCAAagcaaaggtctcacttaagattaactcacacaggagatatcatatgGTGTCCAAAGGGGAAGAGGACAATATGGCATCGTTGCCAGCTACGCATGAACTGCACATCTTCGGCTCGATTAACGGTGTCGATTTCGATATGGTCGGCCAGGGTACGGGGAATCCTAACGACGGTTATGAGGAGCTGAACCTCAAATCGACGAAGGGGGATCTCCAGTTTAGCCCCTGGATTTTGGTCCCACATATTGGTTACGGCTTTCATCAGTACCTCCCGTATCCGGACGGTATGAGCCCTTTTCAAGCTGCTATGGTGGACGGTAGCGGTTACCAAGTCCACCGGACCATGCAGTTTGAGGATGGGGCATCGCTGACGGTCAACTACCGTTATACCTACGAAGGT

pJE483_484_ins gBlock

AAGCAAtctagagacgaacaataaggcctccctaacggggggccttttttattgataacaaaagtcgtggtttgtctggtcaaccaccgcggtctcagtggtgtacggtacaaaccccgacgcggccgcTACAATCGGACTGCGCTGTATAGTCG

pJE990_Block_1

GGGAGACCAGAAACAAAAAAAGGCCGCGTTAGCGGCCTTCAATAATTGGaCCTGGCTCCTaACTGATTTTTAAGGCGACTGATGAGTCGCCTTTTTTTTGTCTaaGAATTCatcagaagaactcgtcaagaaggcgatagaaggcgatgcgctgcgaatcgggagcggcgataccgtaaagcacgaggaagcggtcagcccattcgccgccaagctcttcagcaatatcacgggtagccaacgctatgtcctgatagcggtccgccacacccagccggccacagtcgatgaatccagaaaagcggccattttccaccatgatattcggcaagcaggcatcgccatgggtcacgacgagatcctcgccgtcgggcatccgcgccttgagcctggcgaacagttcggctggcgcgagcccctgatgctcttcgtccagatcatcctgatcgacaagaccggcttccatccgagtacgtgctcgctcgatgcgatgtttcgcttggtggtcgaatgggcaggtagccggatcaagcgtatgcagccgccgcattgcatcagccatgatggatactttctcggcaggagcaaggtgagatgacaggagatcctgccccggcacttcgcccaatagcagccagtcccttcccgcttcagtgacaacgtcgagcacagctgcgcaaggaacgcccgtcgtggccagccacgatagccgcgctgcctcgtcttggagttcattcagggcaccggacaggtcggtcttgacaaaaagaaccgggcgcccctgcgctgacagccggaacacggcggcatcagagcagccgattgtctgttgtgcccagtcatagccgaatagcctctccacccaagcggccggagaacctgcgtgcaatccatcttgttcaatcatgcgaaacgatcctcatcctgtctcttgatcagatcttgatcccctgcgccatcagatccttggcggcaagaaagccatccagtttactttgca

pJE990_Block_2

agatccttggcggcaagaaagccatccagtttactttgcagggcttcccaaccttaccagagggcgccccagctggcaattccggttcgcttgctgtccataaaaccgcccagtctagctatcgccatgtaagcccactgcaagctacctgctttctctttgcgcttgcgttttcccttgtccagatagcccagtagctgacattcatccggGACGTCGTGCCCCAACTGGGGTAACCTTTGAGTTCTCTCAGTTGGGGGatcgatagtcaaaagcctccggtcggaggcttttgactagcacctcggtaccaaattccagaaaagaggcctcccgaaaggggggccttttttcgttttggtccactagtagtcttagtcttcaCCGATGAGCTACCCAGTAGTagaagacaacttaagattaactcacacaggagatatcatatgGTGTCCAAAGGGGAAGAGGACAATATGGCATCGTTGCCAGCTACGCATGAACTGCACATCTTCGGCTCGATTAACGGTGTCGATTTCGATATGGTCGGCCAGGGTACGGGGAATCCTAACGACGGTTATGAGGAGCTGAACCTCAAATCGACGAAGGGGGATCTCCAGTTTAGCCCCTGGATTTTGGTCCCACATATTGGTTACGGCTTTCATCAGTACCTCCCGTATCCGGACGGTATGAGCCCTTTTCAAGCTGCTATGGTGGACGGTAGCGGTTACCAAGTCCACCGGACCATGCAGTTTGAGGATGGGGCATCGCTGACGGTCAACTACCGTTATACCTACGAAGGTAGCCATATTAAGGGCGAAGCTCAAGTGAAGGGTACCGGCTTTCCGGCGGACGGTCCGGTGATGACGAACTCCCTCACCGCCGCCGATTGGTGTCGTAGCAAGAAAACCTATCCGAATGACAAAACCATCATCTCGACGTTTAAGTGGAGCTATACGACGGGTAATGGCAAGCGCTACCGTTCGACGGCACGTACCACCTACAC

pJE990_Block_3

ATGGCAAGCGCTACCGTTCGACGGCACGTACCACCTACACGTTTGCAAAGCCTATGGCTGCAAATTACCTCAAGAATCAACCTATGTATGTGTTCCGGAAAACGGAACTCAAACATAGCAAAACGGAACTGAACTTCAAAGAGTGGCAGAAAGCTTTCACGGATGTCATGGGTATGGACGAACTCTATAAATAAtctagaGACGAACAATAAGGCCTCCCTAACGGGGGGCCTTTTTTATTGATAACAAAAaTCCACaAGGAAAAATTAAAGGGGAGATAAAATCCCCCCTTTTTGGTTAACTgcggccgcGTCGTGGTTTGTCTGGTCAACCACCGCGGTCTCAGTGGTGTACGGTACAAACCCCGACgctagcAACGCATGAGAAAGCCCCCGGAAGATCACCTTCCGGGGGCTTTTTTATTGCGCtgcgggtgccagggcgtgcccttgggctccccgggcgcgtactcc

pJE991_Block_2

agatccttggcggcaagaaagccatccagtttactttgcagggcttcccaaccttaccagagggcgccccagctggcaattccggttcgcttgctgtccataaaaccgcccagtctagctatcgccatgtaagcccactgcaagctacctgctttctctttgcgcttgcgttttcccttgtccagatagcccagtagctgacattcatccggGACGTCGTGCCCCAACTGGGGTAACCTTTGAGTTCTCTCAGTTGGGGGatcgatagtcaaaagcctccggtcggaggcttttgactagcacctcggtaccaaattccagaaaagaggcctcccgaaaggggggccttttttcgttttggtccGGATCCATCCaagtcttcaattgcaatccgataattcaGGTagaagacaaCTGGCTCGAGGACGAACAATAAGGCCTCCCTAACGGGGGGCCTTTTTTATTGATAACAAAAaTCCACaAGGAAAAATTAAAGGGGAGATAAAATCCCCCCTTTTTGGTTAACTgcggccgcGTCGTGGTTTGTCTGGTCAACCACCGCGGTCTCAGTGGTGTACGGTACAAACCCCGACgctagcAACGCATGAGAAAGCCCCCGGAAGATCACCTTCCGGGGGCTTTTTTATTGCGCtgcgggtgccagggcgtgcccttgggctccccgggcgcgtactcc
